# Evaluation of a Dry EEG System for Application of Passive Brain-Computer Interfaces in Autonomous Driving

**DOI:** 10.3389/fnhum.2017.00078

**Published:** 2017-02-28

**Authors:** Thorsten O. Zander, Lena M. Andreessen, Angela Berg, Maurice Bleuel, Juliane Pawlitzki, Lars Zawallich, Laurens R. Krol, Klaus Gramann

**Affiliations:** ^1^Biological Psychology and Neuroergonomics, Technical University of BerlinBerlin, Germany; ^2^Team PhyPA, Biological Psychology and Neuroergonomics, Technical University BerlinBerlin, Germany; ^3^Center for Advanced Neurological Engineering, University of California San DiegoSan Diego, CA, USA

**Keywords:** autonomous driving, passive BCI, EEG, usability, ERP

## Abstract

We tested the applicability and signal quality of a 16 channel dry electroencephalography (EEG) system in a laboratory environment and in a car under controlled, realistic conditions. The aim of our investigation was an estimation how well a passive Brain-Computer Interface (pBCI) can work in an autonomous driving scenario. The evaluation considered speed and accuracy of self-applicability by an untrained person, quality of recorded EEG data, shifts of electrode positions on the head after driving-related movements, usability, and complexity of the system as such and wearing comfort over time. An experiment was conducted inside and outside of a stationary vehicle with running engine, air-conditioning, and muted radio. Signal quality was sufficient for standard EEG analysis in the time and frequency domain as well as for the use in pBCIs. While the influence of vehicle-induced interferences to data quality was insignificant, driving-related movements led to strong shifts in electrode positions. In general, the EEG system used allowed for a fast self-applicability of cap and electrodes. The assessed usability of the system was still acceptable while the wearing comfort decreased strongly over time due to friction and pressure to the head. From these results we conclude that the evaluated system should provide the essential requirements for an application in an autonomous driving context. Nevertheless, further refinement is suggested to reduce shifts of the system due to body movements and increase the headset's usability and wearing comfort.

## Introduction

Driving has become a part of everyday life, which makes the drive to work or for recreational activities a simple routine task. However, the effects of the mental workload and effort required by driving often go unnoticed. A study by Borghini et al. (2014) found that mental workload, fatigue, and drowsiness are significantly increased while driving. Especially long periods of constant driving, as often performed by professional truck drivers, result in an accumulation of these effects over time, decreasing the driver's cognitive capabilities and driving performance, thus increasing the chances of traffic accidents.

The field of automotive human factors and ergonomics is concerned with minimizing safety risks depending on human performance in driving tasks. Today, many automations and small devices have found their way into cars in order to help reduce the mental workload required to operate the vehicle (Young and Stanton, 1997; Tadaka and Shimoyama, 2004; Ma and Kaber, 2005). A different approach aims to fully or at least partly automate the task of driving, so the human driver can be eliminated as a risk factor in most instances. The scientific field working toward this goal is called *Autonomous Driving* (Franke et al., 1998) and has grown more important over the past years.

One particular problem with autonomous driving is the question of responsibility: Who is accountable in case of an accident? Most countries still define the human driver of a car as the entity responsible for anything that happens while driving (Beiker, 2012). Therefore, experts believe it would be best to only automate some of the tasks that arise while driving, but to leave the most complex tasks to a human driver for the time being. According to Sukthankar et al. (1997), the task of driving consists of different levels, which are the strategic level (route planning), the tactical level (maneuver selection), and the operational level (maneuver operation). Automation of the lowest, operational level is thus legally the least complex, and also technically possible (Dickmanns and Zapp, 1987; Pomerleau, 1992). Driving along a highway could relatively easily be automated, but once the traffic situation changes, the human may be required to take over control. This approach thus requires an important exchange of information between the human driver and the automated system: The human must be timely and appropriately informed of the pending takeover. As stated by Llaneras et al. (2013), people tend to focus their attention on secondary tasks once the primary objective of driving has been taken over by automation. As a consequence, in a situation where the car drives autonomously, a signal given by the system to indicate the necessity for takeover might be missed, or might catch the human by surprise. This may result in loss of control over the vehicle.

As a solution to the above problem, the car could monitor the driver's mental state, and adapt the notification process to the current context. A completely attentive driver might quickly perceive and understand even simple signals, whereas for example a sleeping driver may need to be woken carefully by the car in advance of leaving the highway. *Passive brain-computer interfaces* (passive BCIs, Zander and Kothe, 2011) are promising approaches for such monitoring and automated adaptation (Zander et al., 2011). This technology enables real-time detection of mental conditions like fatigue, workload, and degree of relaxation (Blankertz et al., 2010; Gerjets et al., 2014), which offer a good estimate of whether or not the driver is ready to take over control of the car. But the passive BCI approach during autonomous driving is not limited to this. More general information—like mood or situational awareness—and also very specific information about the subjective interpretation of the current context—that might be reflected in the driver's brain as error responses—could be assessed by the passive BCI (Zander and Jatzev, 2012). This information could then be integrated in the autonomous decisions of the car. The car learns how the driver interprets the context and gains a degree of context-awareness by utilizing the driver's brain as a sensor.

Passive BCIs are commonly based on electroencephalography (EEG). Traditional EEG systems are relatively cumbersome to apply and use, requiring preparation of the skin, application of conductive gel, and cleaning of the cap afterwards. To make EEG applicable for non-scientific uses, e.g., to be used by drivers, its application and handling needs to be as easy as possible. This is why alternative electrode systems (e.g., described in Zander et al., 2011; Liao et al., 2012) are an important focus of autonomous driving related BCI research. Primarily, the use of gel is eliminated, and the caps containing the electrodes are made for quick application, resulting in less preparation time and, ideally, more comfortable for the wearer. Recent laboratory studies provided evidence of good signal quality, comparable to that of standard gel-based electrodes. It is still unclear however that the signal quality can be maintained in real-world contexts.

This study focused on evaluating the use and application of a dry electrode EEG system in the context of a running vehicle. It was assessed how easy it is for untrained person to apply the system on their own head, how well the electrodes can be positioned and remain in place, and whether the signal quality is sufficient for BCI usage when the system is self-applied. Two common features in the EEG, an N200-P300 ERP and the parietal alpha rhythm, were analyzed as examples of signals that potentially can be used in a passive BCI application. Furthermore, interference in the EEG signal resulting from usage inside a running car—a noisy environment—was investigated. Finally, wearing comfort over a prolonged period of time as well as general user acceptance were evaluated.

## Materials and methods

### Participants

Ten participants, five male, participated in the experiment. The mean age was 28 years (*SD* = 3.4). Two participants reported to have sensitive skin. All participants gave their written informed consent to participate in the study and were paid 20 euros as expense allowance. The overall duration of the experiment was on average 165 min (*SD* = 39 min.), including breaks.

### Apparatus

#### Vehicle

The vehicle we used to evaluate the influence of vehicle-induced noise on the recorded EEG was a Volkswagen Touran (year of manufacture 2003). The car was stationary during the experiments, but had the engine running, the radio switched on (though muted), and the air conditioning enabled. A 7.6″ TFT-display was mounted to the right of the steering wheel near the center console.

#### Experimental room

The experimental room used for baseline recordings was a non-frequented room at the TU Berlin with constant light, right next to the parked car. Diversions and disturbances were kept to a minimum.

#### Computer system

The EEG system was connected to a laptop (Sony Vaio Z, 2012) and EEG data was recorded using the *BrainVision Recorder, BrainVision RDA* (Brain Products GmbH, Munich, Germany), and *LabRecorder* (as part of the BCILAB framework, Delorme et al., 2010). The experimental paradigms were run using *SNAP*^1^ (Iversen and Makeig, 2013). To analyze the data, we used the EEGLAB toolbox (Delorme and Makeig, 2004), an open source toolbox embedded in MATLAB. For classification we used the open source toolbox BCILAB (Kothe and Makeig, 2013), also embedded in MATLAB.

#### EEG system

**Figure 1:**
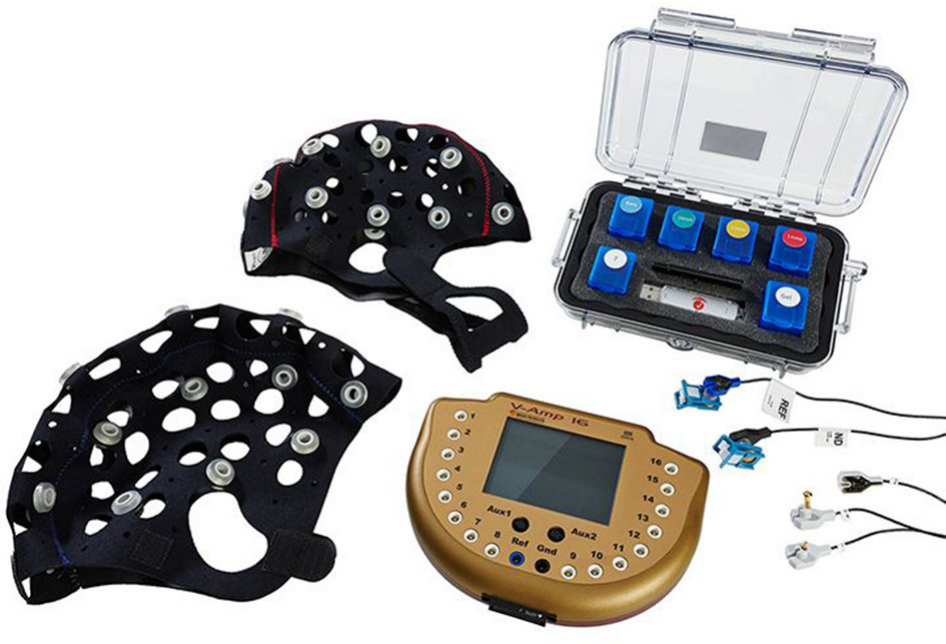
**Overview of the used EEG system, the Brain Products actiCAP Xpress**. Image courtesy of Brain Products GmbH.

The system examined in this study was the *Brain Products actiCAP Xpress* dry-electrode EEG system (see Figure 1) provided by Brain Products GmbH for the duration of the experiment. The system included 16 active data electrodes plus one reference and one ground electrode. Electrodes were applied to one of two differently-sized flexible caps, depending on the head circumference of the participant (52–58, or 58–64 cm). To ensure fixation on the participant's head, a chin belt was attached to the cap. Each cap provided 78 possible electrode positions most of the extended international 10% system, with additional options to set up regions of interest. We used electrode positions Fp1, Fp2, Fz, FC5, FC6, C3, C4, Cz, CPz, Pz, CP5, CP6, PO3, PO4, POz, and Oz.

**Figure 2:**
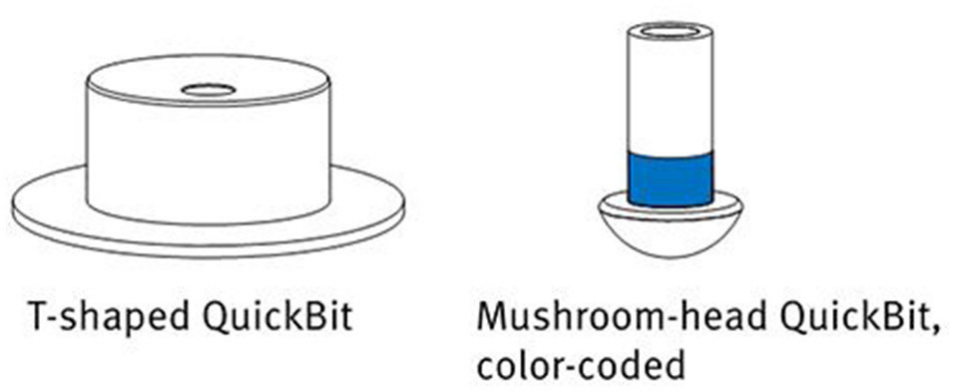
**The different QuickBit types provided with the actiCAP Xpress**. Image courtesy of Brain Products GmbH.

To adjust the system to an individual participant, the electrodes can be extended to different shapes and sizes by attaching so-called *QuickBits* (see Figure 2). The kit used in the study came with six T-shaped flat tips (with a diameter of 7 mm) to be attached to the forehead and earlobes, as well as 32 mushroom-head tips for application on the scalp. These latter come in different lengths of 8, 10, 12, and 14 mm, which can be attached to the electrodes according to head shape and required pressure. This enabled a personalization of the system: Optimal sensor lengths for electrode positions can be noted, stored and re-applied in follow-up experiments.

Prior to applying the *actiCAP Xpress*, the electrodes were cleaned using a disinfectant spray. This was done even in case the electrodes and sensors had not been used before to remove dust and particles to improve connectivity.

The electrode array was connected to a *V-Amp* EEG signal amplifier (Brain Products GmbH, Munich, Germany), which in turn was connected to a laptop computer through a universal serial bus (USB) 2.0.

### Experimental procedure

#### Experimental rationale

This study was designed to assess different requirements to an EEG system for application in real-world driving scenarios. We defined the following requirements: (1) self-applicability of the system, (2) impact of interfering noise signals inside a running vehicle on EEG signal quality, (3) stability of cap and electrode positions after context-related movements, and (4) usability and wearing comfort of the system.

The experiment was divided into four blocks covering these four issues, answering the following questions.

1. How easy and accurate is self-application of the system in comparison to application by another person? (Block I)
2. How strong is the effect of interfering signals in a running car on EEG recording? (Block II)
3. How do electrode positions change during typical body movements inside a car? (Block III)
4. How do participants rate the system's usability? (Block IV)

**Figure 3:**
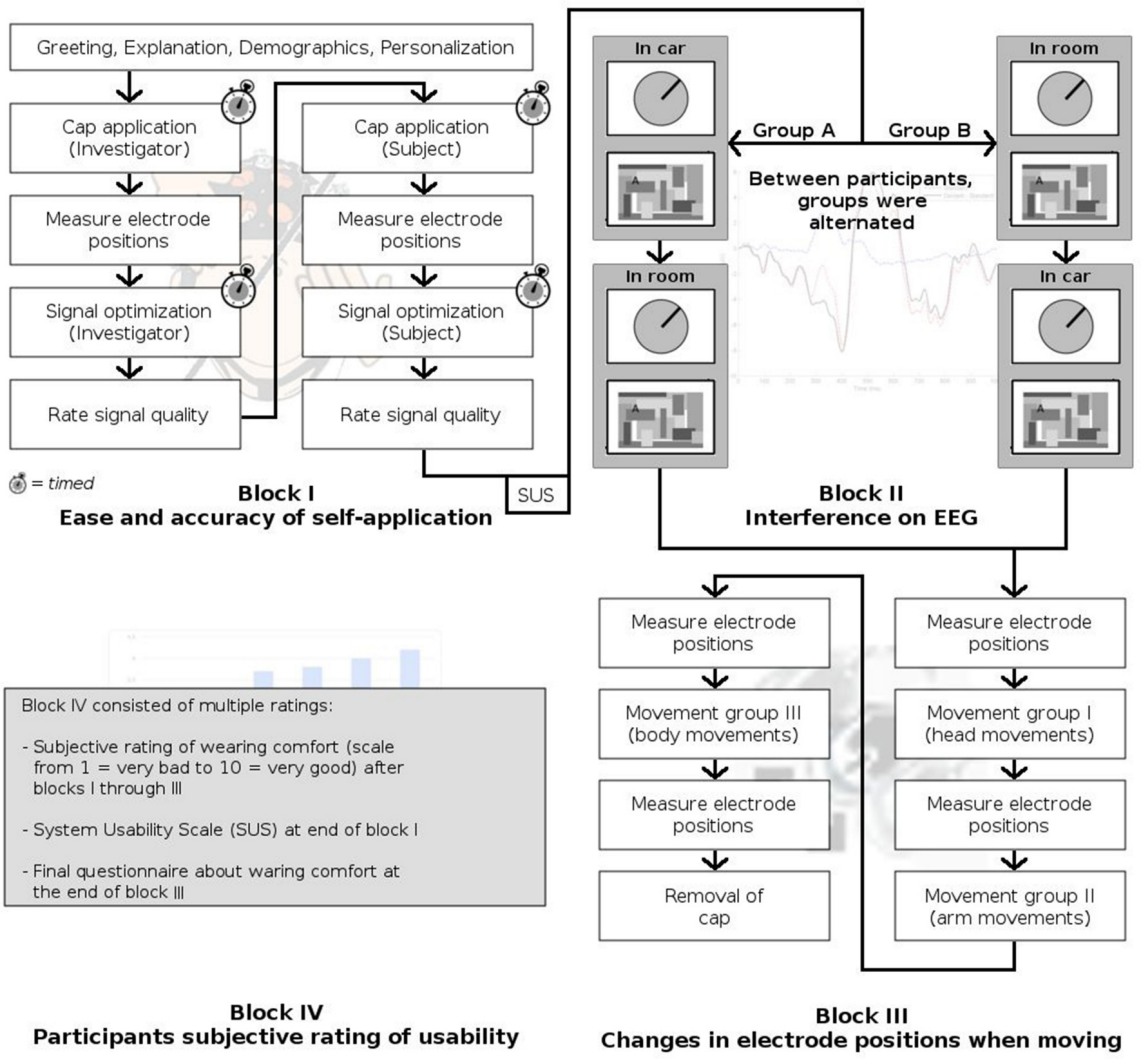
**Experiment timeline**.

Figure 3 summarizes the experimental session. After arrival of the participant, the experiment was explained and a demographic survey was conducted. While the cap was personalized by the investigator by exchanging electrode tips where necessary, the participant was asked to read the instruction manual of the system, in preparation for Block I.

#### Block I: self-application

Self-application of the cap, as opposed to having the cap fitted to you by a trained operator, may take a different amount of time and may affect the positioning of the electrodes and the signal quality. To estimate these effects, we compared cap application in two conditions: Application by the experimenter, and self-application by the participant. Customization of the cap was not included here, as it is assumed to be a one-time effort.

Participants were seated in the experimental room, in front of a laptop. A stopwatch was used to first measure the time required by the experimenter to apply the EEG cap to the participant's head.

Once the cap and ground/reference electrodes were in place, electrode positions were measured using the *Polaris Vicra* system (Northern Digital Inc., Waterloo, ON, Canada), allowing for measuring 3-dimensional electrode locations. We chose to record the 16 electrode positions, as well as the inion, the nasion and the left and right preauricular points. The latter three were used as coordinate references to allow the transformation of coordinates taken from different measuring sessions into one coordinate system to allow comparison (described below in the section “Analysis Procedures”). To achieve comparable, stable positions for the reference points in each measurement during the experiment, we marked them by a small dot on the respective positions on the participant's skin using a removable eudermic marker.

**Figure 4:**
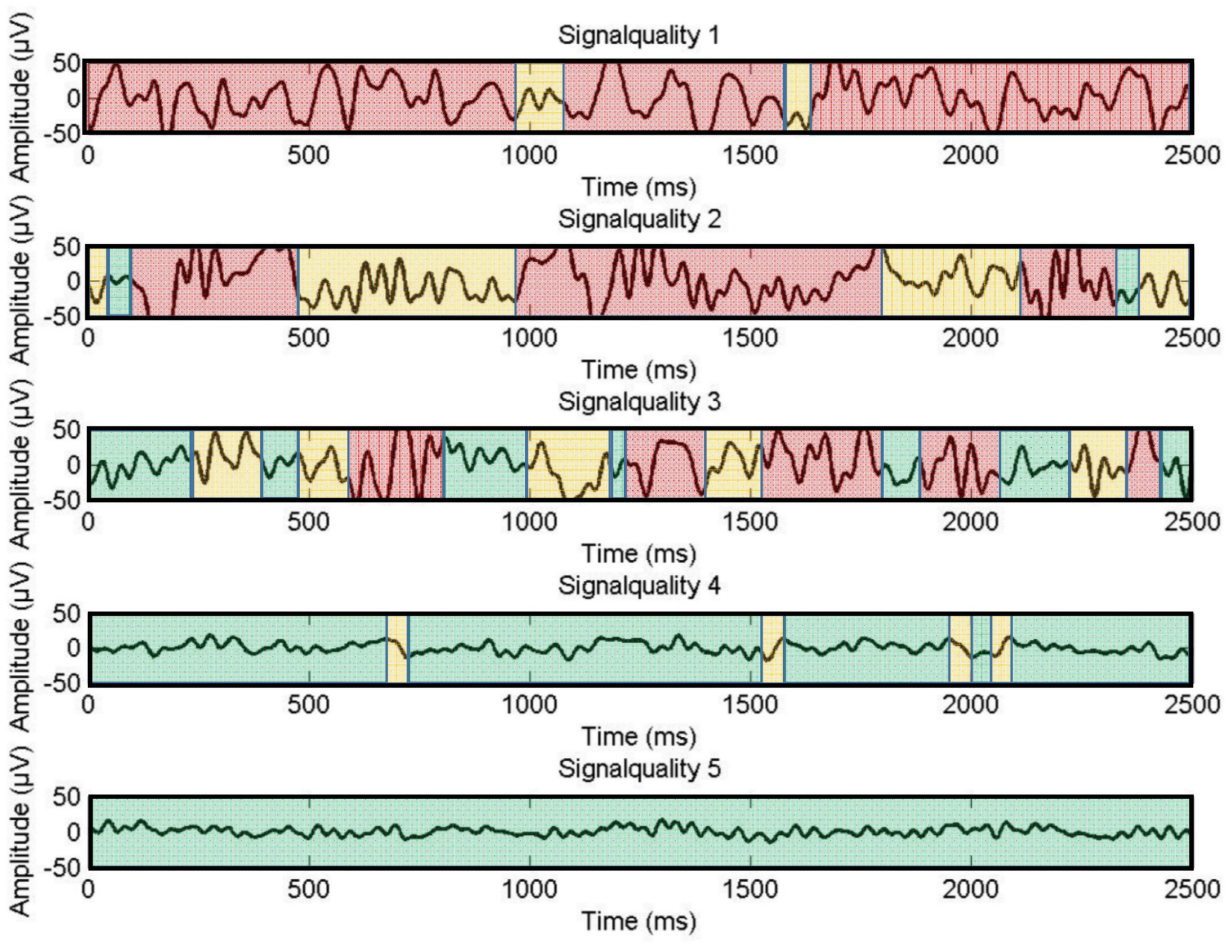
**Examples for signal quality ratings on a scale from one to five**. Green colored parts indicate adequate signal quality, yellow parts moderate signal quality, and red parts unacceptable signal quality.

Following this, signal quality was optimized by relatively fine-grained adjustments to the electrodes. As the system did not provide an objective measure of signal quality or electrode contact (e.g., impedance), signal quality was assessed visually. The signal was monitored using the *BrainVision Recorder* software, with all 16 channels displayed at once, set to a resolution of 50 μV. A *display filter* was enabled, bandpass-filtering the visible signal from 0.1 to 40 Hz, not affecting the recording. The duration of this optimization was again timed using a stopwatch. The resulting signal quality was also recorded, as rated by the experimenter. The indication for signal quality was the visual form of the signal on the display, artifacts had to be recognized visually. The rating followed predefined guidelines and was done on a 5-point scale with 5 meaning “perfect signal” and 1 meaning “no signal at all” (see Figure 4). This rating was done twice: Once for the signals with the display filter switched on, and once based on the unfiltered raw signal.

Following this, the cap was removed and participants, who read the instructions manual, were asked to put on the cap by themselves, after all of their questions about the procedure had been answered by the experimenter. Application time was again measured, as were the electrode positions and the resulting signal quality.

#### Block II: EEG recording

For investigating signal quality in standard EEG analyses we chose the well-known N200 and P300 components of the visual event-related potential and the parietal alpha rhythm. Both time- and frequency domain parameters are well-examined phenomena in EEG research. Hence, clear expectations about morphology, topography and signal strength can be drawn, that build the baseline of comparison for our results.

In order to assess the EEG signal and the possible influence on it of the electromagnetically noisy environment that is the car, participants performed in two established experimental paradigms of BCI research (Zander et al., 2011), once in the experimental room, and once inside the car. The order of these two conditions was randomized between participants.

**Figure 5:**
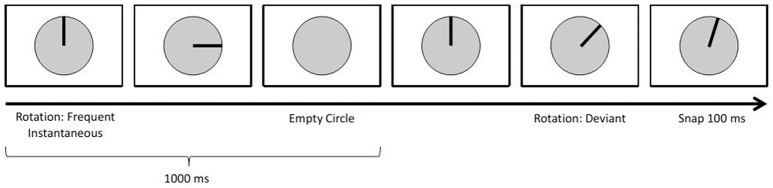
**Oddball Paradigm**.

The first paradigm focused on the elicitation of visual event-related potentials (ERPs) using a standard oddball approach: An infrequent *deviant* stimulus sometimes appeared instead of the frequent *standard* stimuli (Duncan-Johnson and Donchin, 1977; see Figure 5). This is a common approach when researching ERPs referred to as the N200-P300 complex (Polich and Kok, 1995; Linden, 2005). ERP detection during autonomous driving can be useful, as they may allow a car to detect how drivers react cognitively to perceived stimuli/information.

On the screen, participants saw a circle divided by lines into 30° angles. First, a bar appeared, like a clock's arm pointing 12 o'clock. This bar then rotated clockwise in discrete steps, once every second. A standard stimulus had it rotate by 90°; a deviant consisted of an initial 60° rotation, followed by a 100 ms pause and a 15° counterclockwise rotation. After each deviant, the bar disappeared and reappeared at the 12 o'clock position.

10% of all stimuli were deviants. In total 400 trials were displayed (360 standard, 40 deviant).

The second paradigm focused on features in the spectral domain, specifically the parietal alpha rhythm. This feature is of special interest to autonomous driving, as parietal alpha can be used as an indicator of whether the participant is currently in a relaxed state or performing some mentally demanding task (Berka et al., 2007). It also is a standard example for features in the spectral domain.

**Figure 6:**
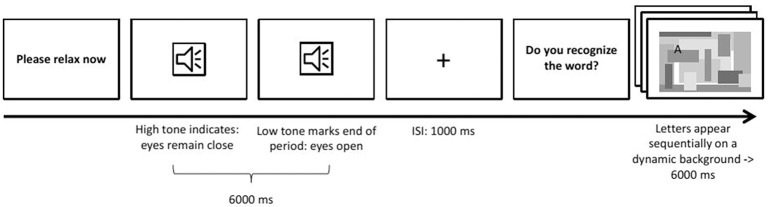
**Induced Alpha Paradigm**.

The paradigm (see Figure 6) presented to the participant was designed to induce changes in parietal alpha activity by alternating between two states of mind: *Engaged* and *relaxed*. To engage the participant, a six-letter word was presented letter by letter, with letters appearing on random locations on the screen amidst visual noise. Each letter was only visible for 1 s. Participants were instructed to read the word. After each engagement trial, the participant was instructed simply to relax for 6 s with their eyes open. This relaxation phase was introduced using an auditory signal and ended by a similar one with lower pitch.

There were 32 trials of each condition. The order of words in the engaged condition was randomized across participants.

These two paradigms were presented in fixed order to the participants in the two conditions (room vs. car).

#### Block III: driving-related movements

The third block investigated the influence of movements on the position of the electrodes.

Electrode positions were recorded, using again the Polaris system mentioned earlier, at the start of this block. Participants then performed a series of three different types of driving-related movements inside the car, and the electrode positions were measured again after each group of movements. Because measurements were not done inside the car but in a nearby room, some walking was required. Electrode cables were bundled together and fixed to the participant's clothing in a relaxed way to minimize their strain on the cap while walking.

To make movements comparable between participants, we placed markers (sticky notes) at certain places in the car: One on the left rear window, one above the driver's seat, one in the legroom of the front passenger seat and one in the center of the rear bench seat. Before seating the participant in the driver's seat, the markers were shown to them. The EEG system was not connected to the amplifier during the movements. All instructions for different movements were given through pre-recorded audio files played back using a laptop and speakers inside the car.

#### Block IV: usability

To assess the usability of the system, the participants were asked to fill out a questionnaire right after Block I. This questionnaire was the *System Usability Scale* (SUS; Brooke, 1986) was employed, also used in other BCI related studies prior to this one (Pasqualotto et al., 2011; Duvinage et al., 2012). SUS is a standardized questionnaire consisting of ten questions based on Likert scales with five options ranging from “strongly disagree” to “strongly agree.” In total, SUS contains five positively and five negatively formulated questions about the system being assessed, for example “I think that I would like to use this system frequently” or “I found the system unnecessarily complex.” From the answers given, a SUS score is calculated, ranging between 0 (worst possible system) and 100 (best possible system). This score has to be interpreted taking the individual context of system usage into account. In contrast to qualitative assessments, the SUS does not yield any insight into which usability problems exactly are present within the system. It provides however a quick and reliable way to determine whether or not major changes are necessary in order to make the system safe and comfortable to use.

Additionally, the participants were asked to rate the level of comfort wearing the system after each of the previously described experimental blocks (I–III) on a scale from 1 to 10, one meaning “extremely bad” and ten “very comfortable.” We acquired these three subjective impressions to gather insight into how the system's perceived comfort changed over the course of the experiment.

To get an even deeper insight into the comfort of wearing the system, participants were asked to fill out another questionnaire after the third experimental block, after roughly 140 min of wearing the system almost constantly. We adapted a questionnaire for the evaluation of the wearing comfort for firemen helmets (Fabrizio and Cimolino, 2014), by only keeping questions deemed fitting to our context. All questions were rated on a five point Likert scale. In addition to these questions, we asked two yes-no questions: Whether or not the participant believed the cap had moved, and whether or not it induced the feeling of dents on their head. Finally, we asked the participants to mention any discomfort associated with wearing the system, like the feeling of pressure on the head, headaches, or nausea.

### Analysis procedures

#### Block I: self-application

Comparison of time needed by the experimenter and the participant to apply the system and to adjust the electrodes was done by two-sample *t*-tests.

The signal quality ratings were subjected to a three-way mixed measures ANOVA with the two within-subject factors visual filters (no filters vs. 0.1–40 Hz bandpass) and electrode (Fp1 vs. Fp2 vs. vs. Fz vs. FC5 vs. FC6 vs. C3 vs. C4 vs. Cz vs. CPz vs. Pz vs. CP5 vs. CP6 vs. PO3 vs. PO4 vs. POz vs. Oz) and the between-subject factor applicant (investigator vs. participant).

Because a total of six different measurements of electrode positions were taken during the course of this experiment, these measurements were first transformed into one coordinate system to allow a unified comparison. To this end, all measurements were re-referenced to a mean head middle and radius, within participants, as follows.

1. All coordinates of recording *j, j* = 1, …, 6 were referenced to the head midpoint *hm*_*j*_, which is calculated with the recorded reference points (nasion *n*_*j*_ and left and right preauricular points, *lp*_*j*_ and *rp*_*j*_) by
  - Drawing a line through both preauricular points *lp*_*j*_ and *rp*_*j*_:
    Calculate the slope by computing new coordinates
      $$\begin{array}{l} {\left(u_{j}\right)}_{i}:={\left(lp_{j}\right)}_{i}-{\left(rp_{j}\right)}_{i},\text{for }i=1,2,3\text{ denoting the}\\ \text{scalars of the three}-\text{dimensional vector }u_{j}. \end{array}$$
    Define the line by
      $$\begin{array}{l} g_{j}\;:=lp_{j\;}+\;r_{j}u_{j}\text{ with }r_{j}\text{ to be determined}. \end{array}$$
  - Construction of a plane *H*_*j*_ through *n*_*j*_, which is perpendicular to the line *g*_*j*_:
    Find the variables *x, y, z* to determine the plane equation for *H*_*j*_
      $$\begin{array}{l} H_{j}:{\left(u_{j}\right)}_{1\;}x\;+{\left(u_{j}\right)}_{2}y\;+\;{\left(u_{j}\right)}_{3}z\;:=e. \end{array}$$
    To find *e*, insert the coordinates of the nasion reference point *n*_*j*_ into the equation
      $$\begin{array}{l} H_{j}(n_{j}):\;{\left(u_{j}\right)}_{1\;}{\left(n_{j}\right)}_{1}+{\left(u_{j}\right)}_{2\;}{\left(n_{j}\right)}_{2}+{\left(u_{j}\right)}_{3\;}{\left(n_{j}\right)}_{3}=e. \end{array}$$
  - For the purpose of finding the intersection of the line *g*_*j*_ with the plane *H*_*j*_, insert the coordinates of *g*_*j*_ into the plane equation above and solve for *r*_*j*_:
    $$\begin{array}{l} H_{j}\left(g_{i}\right):r_{j}=\frac{e-{\left(u_{j}\right)}_{1}{\left(lp_{j}\right)}_{1}-{\left(u_{j}\right)}_{2}{\left(lp_{j}\right)}_{2}-{\left(u_{j}\right)}_{3}{\left(lp_{j}\right)}_{3}}{{\left(u_{j}\right)}_{1}^{2}+{\left(u_{j}\right)}_{2}^{2}+{\left(u_{j}\right)}_{3}^{2}}. \end{array}$$
    Inserting *r*_*j*_ into the plane equation yields the head midpoint:
      $$\begin{array}{l} hm_{j}=lp_{j}+r_{j}u_{j}. \end{array}$$
2. After calculating the head midpoints *hm*_1_ to *hm*_6_, we compute the arithmetic average $\bar{hm}$ over all recordings as the final reference point in order to minimize the error of measurement in the system.
3. The deviation of the recorded head midpoint *hm*_*j*_ to *hm* is calculated for each recording:
  $$\begin{array}{l} d_{j}\;:=hm_{j}-\bar{hm}\;,\;\;j\;=1,\ldots ,6. \end{array}$$
4. Then, all recorded electrode positions (*e*_*p*_*k*_)*j*_, *k* = 1, …, 16 are re-referenced to *hm* by addition with *d*_*j*_ and the euclidean distance *ed*_*j*_1_*j*_2__ between different recordings *j*_1_, *j*_2_ is calculated:
  $$\begin{array}{ll} {\left(d_{j_{1}j_{2}}\right)}_{i}\; & :={\left({\left(ep_{k}\right)}_{j_{1}}+d_{j_{1}}\right)}_{i}-{\left({\left(ep_{k}\right)}_{j_{2}}+d_{j_{2}}\right)}_{i},\\ ed_{j_{1}j_{2}} & :=\sqrt{{\left(d_{j_{1}j_{2}}\right)}_{1}^{2}+{\left(d_{j_{1}j_{2}}\right)}_{2}^{2}+{\left(d_{j_{1}j_{2}}\right)}_{3}^{2}} \end{array}$$

The value used for comparison of different recordings *j*_1_, *j*_2_ was this euclidean distance *ed*_*j*_1__, _*j*_2__.

For Block I, recorded positions from the investigator-applied cap were compared to the positions from the self-applied cap. Mean differences of electrode positions were then compared to the expected value of no difference in positions using a one-sample *t*-test against zero.

#### Block II: EEG recordings

##### Oddball paradigm: ERP analysis

EEG data was first preprocessed by applying a bandpass-filter from 1 to 30 Hz, retaining all frequencies relevant for later analyses. Then, epochs of 1100 ms were extracted, starting 100 ms before stimulus onset of the standard and deviant events. Baseline correction was performed with a 100 ms pre-stimulus interval.

To compare event-related activity between car and indoor recordings, amplitudes and latencies of the N200's and P300's were extracted.

First, the indoor condition was used as a baseline as it conforms to laboratory conditions. Inspection of the grand average revealed a global negative minimum at 300 ms over the centro-parietal lead (Pz) and a global positive maximum at 400 ms over the centro-central lead (Cz). Based on these peaks, a search window was defined around 300 ± 70 and 400 ± 70 ms to search for maxima in the individual averages. Once for each individual the global peaks were identified, the peaks on individual channels were identified using a ± 25 ms window around the individual global peak. Mean amplitudes and latencies were extracted for all channels. This procedure resulted in a 4 x 16 vector for each participant, consisting of the mean amplitudes and the latencies of the two components at each channel.

For comparison of mean peak amplitudes two repeated measures ANOVAs were performed. Mean amplitudes from electrode Pz were used for the negativity and from Cz for the positivity. Each 2x2 ANOVA had the two within-participant factors recording condition (indoor vs. car) and stimulus (standard vs. deviant).

In order to examine disparities of mean peak latencies between conditions (indoor vs. car), mean difference peak latencies were calculated by subtracting the negative from the positive peak latency. The mean difference was taken per participant for the two conditions and subjected to a paired sample *t*-test.

To test for equivalence of EEG measures between recording conditions the two one-sided tests (TOST, Schuirmann, 1981, 1987; Westlake, 1981) procedure was applied to mean peak amplitudes and mean difference peak latencies with an epsilon of the standard deviation of the indoor condition, which was regarded as the control group (R-package “equivalence” May 14, 2016; V0.7.2). A *p*-value of 0.05 was taken as the significant threshold for all TOST.

##### Induced alpha paradigm: frequency analysis

To compare oscillatory features between car and indoor recordings, three different measures were taken: The power spectral density function covering 0.1–40 Hz, single measurements of the band power in the alpha band, and the time course of the alpha band power during the 6-s trials of the paradigm (engaged vs. relaxed).

Fluctuations in alpha power occur with a broader distribution over posterior areas of the scalp (Sauseng et al., 2005). Since we were interested in parietal alpha as potential indicator of mental load, analyses were restricted to five posterior electrodes, namely Pz, PO3, PO4, POz, and Oz. The data was bandpass filtered from 0.1 to 40 Hz and time epochs of 6 s were selected, covering each full trial.

Power spectral densities (PSD) were calculated for each entire epoch and averaged per participant, resulting in 2 x 2 x 5 PSD distributions for each participant (2 experimental conditions x 2 mental states x 5 channels). We used these participant-individual PSDs as well as the averaged PSDs over all participants (grand average), resulting in a total of 11 (2 x 5+1) PSD-distributions for each experimental condition.

Individual and grand average Pearson Correlation of the PSD in the frequency band of 0.1 Hz to 40 Hz were calculated for each electrode between indoor and car conditions and tested for significance using one sample *t*-tests against zero.

The alpha band (7–13 Hz) being of prime interest here, we also calculated a single bandpower value in this frequency range for each participant, electrode, and trial. We used epochs of 4 s length, starting 2 s after stimulus onset. Logarithmic variances of each trial per electrode of each participant were calculated and normalized with the maximum value of each electrode. These measures were then averaged over all trials, resulting in a normalized mean alpha band power for each participant under each experimental condition on the five investigated electrodes. Effects between recording conditions, stimuli and electrodes were investigated in a 2 x 2 x 5 ANOVA with the three within-participant factors recording condition (indoor vs. car), stimulus (standard vs. deviant) and electrode (Pz vs. PO3 vs. PO4 vs. POz vs. Oz). The factor electrode is a repeated measure here as EEG measures at one electrode depend on values measured by other electrodes. Again, the TOST procedure with an epsilon of the standard deviation of the indoor condition was applied to normalized mean alpha band power values to test for equivalence between recording conditions.

As a third measure, the time course of the band power in the alpha band was used. It was calculated by shifting a 500 ms window over each single trial and calculating the band power for each window position. To avoid leakage effects, the window was multiplied with a Gaussian bell curve of the same size. Afterwards the single-trial measurements were normalized with the mean of all band powers. The normalized measurements were averaged, resulting in 2 x 5 time courses for each participant (2 experimental conditions x 5 channels). As above, we also took the grand average into account, resulting in 11 time courses in total per experimental condition.

To examine the difference in the time course of the band power in the alpha range between conditions, Pearson Correlations were calculated for each participant, channel and condition.

##### BCI Analysis of both paradigms

BCILAB's built-in classification approaches were used to evaluate the offline single-trial accuracies as an estimate of potential online performance.

For the oddball paradigm, data was bandpass filtered from 0.1 to 15 Hz and downsampled to 100 Hz. Epochs of 800 ms were extracted starting at each stimulus marker. A windowed-means approach (Blankertz et al., 2011) was used to extract features, using 8 consecutive windows of 50 ms starting at 300 ms post-stimulus. As a classifier we used *linear discriminant analysis*, LDA (Webb, 2002). Mean ERP classification error rates of all eight participants were subjected to a paired samples *t*-test.

*Logarithmic band power* was used for feature extraction (Solis-Escalante et al., 2010; Zander et al., 2011) of the data of the second paradigm. This was applied to epochs of 6 s, as above. We performed a (10 x 10)-fold cross-validation, and classified using LDA. Mean classification error rates were subjected to a paired samples *t*-test.

Classification error rate results from both paradigms were subjected to a TOST procedure with an epsilon of the standard deviation of the indoor condition to test for equivalence between recording conditions.

#### Block III: driving-related movements

Each of the three movement groups had one electrode position measurement before, and one after it. Mean differences of electrode positions prior to and after each movement group were compared to the expected value of no difference in positions using a one-sample *t*-test against zero.

#### Block IV: usability

The *System Usability Scale* was interpreted following the guidelines set by Brooke (1986). To determine the resulting SUS score of the system, all given answers were weighted accordingly and added up. This resulted in a total score per participant, which then was multiplied by the factor 2.5.

After experimental blocks I to III, participants were asked to give a subjective estimate of how comfortable the system felt. The median of the comfort ratings of all participants was used as the overall comfort rating here. To test for differences between the three time points, a Wilcoxon Signed-rank test was applied. The wearing comfort questionnaire was evaluated descriptively.

## Results

### Block I: self-application

#### Application time

A two-samples *t*-test indicated that the mean time needed for application of the cap did not differ significantly between experimenter (*M* = 123.2 s, *SD* = 43.8) and participants (*M* = 104.9 s, *SD* = 49.0), *t*_(9)_ = 0.880, *p* = 0.391, though showing a tendency that participants perform faster. Mean times needed for adjustment of electrodes also did not differ significantly between investigator (*M* = 256.3 s, *SD* = 221.3) and participants (*M* = 310.2 s, *SD* = 285.1), *t*_(9)_ = 0.472, *p* = 0.642, showing a tendency that experimenters are faster.

#### Electrode signal

The three-way mixed measures ANOVA on signal quality ratings revealed no significant main effect of applicant, *F*_(1, 18)_ = 0.341, *p* = 0.341, η^2^ = 0.019. The main effect of filter was significant, *F*_(1, 18)_ = 66.861, *p* = 0.000, η^2^ = 0.788. Since the main effect of electrode violated the assumption of sphericity Greenhouse-Geisser corrected values were used. The main effect electrode was significant, *F*_(5.167, 93.012)_ = 2.876, *p* = 0.017 η^2^ = 0.138. None of the interaction effects were significant, all *ps* > 0.281.

#### Electrode positions

**Figure 7:**
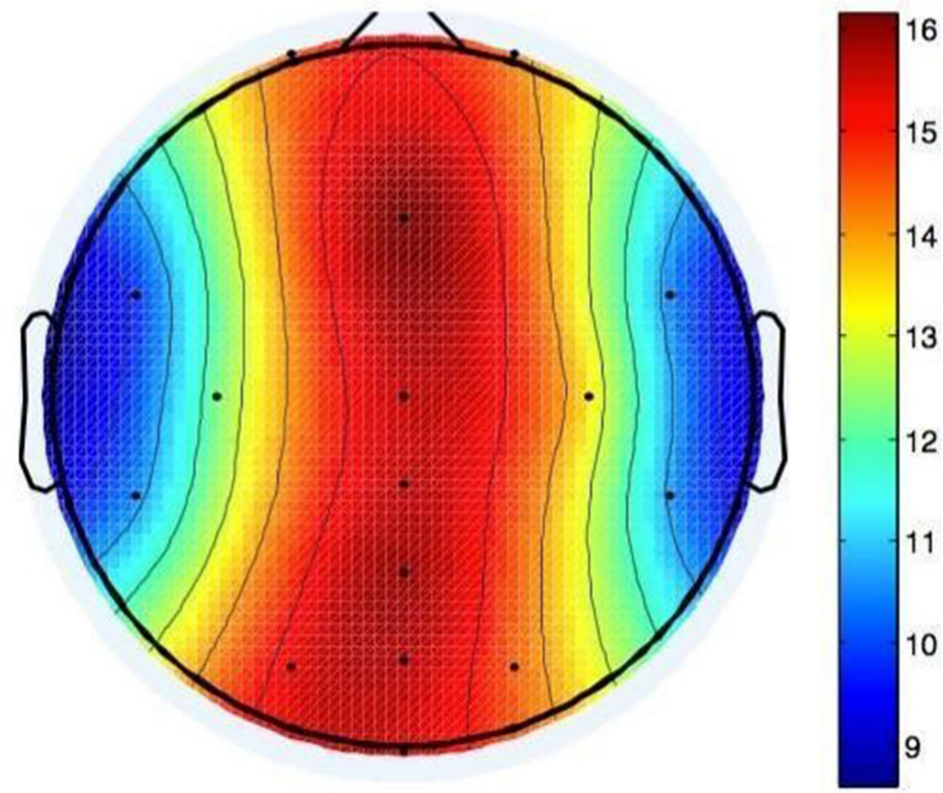
**Shifts in electrode positions after self application in mm compared to application by investigator**.

The *t*-test against zero performed on mean differences of electrode positions (*M* = 13.76 mm, *SD* = 5.12 mm) between investigator- and self-applied cap yielded significance, *t*_(9)_ = 8.498, *p* = 0.00001. The electrode positions varied most on the midline of the head, with 15.5 mm variation (averaged over all 10 participants) at Oz to 16.1 mm averaged variation at Fz. This could be due to the structure of the cap: It has two holes for the ears, so electrodes in this area are fixated more clearly than electrodes elsewhere. Electrodes on the forehead can be positioned up to 1 cm higher or lower without any obvious effects on the cap like inconvenience or ill-fittingness, so it was hard for both participants and investigators to position the cap correctly around the midline of the head (see Figure 7).

For Block I, recorded positions from the investigator-applied cap were compared to the positions from the self-applied cap. Mean differences of electrode positions were then compared to the expected value of no difference in positions using a one-sample *t*-test against zero.

### Block II: EEG recordings

Due to software problems on a laptop EEG data of two participants had to be excluded. Analyses of the EEG data were based on the remaining eight participants.

#### Oddball paradigm: ERP results

**Figure 8:**
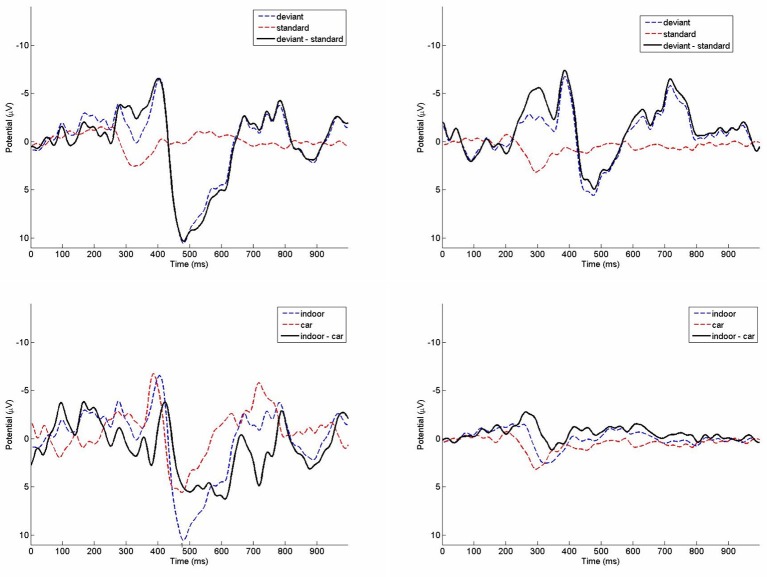
**Grand average ERPs of the indoor condition (top left)** and the running car condition **(top right)** on channel Cz. Deviant **(bottom left)** and standard **(bottom right)** ERPs in comparison between indoor and car condition.

Grand average ERPs from the oddball paradigm are depicted in Figure 8. The repeated measures ANOVA performed on mean amplitudes of the negativity measure yielded significance for the main factor stimulus, *F*_(1, 7)_ = 21.745, *p* = 0.002, η^2^ = 0.756. Amplitudes of the deviant stimuli (*M* = −5.44 μV, *SD* = 6.21 μV) were more negative than in standard stimuli (*M* = −0.01 μV, *SD* = 2.66 μV). The main factor environment was not significant, *F*_(1, 7)_ = 0.101, *p* = 0.760, η^2^ = 0.014. There was also no significant interaction, *F*_(1, 7)_ = 0.261, *p* = 0.625, η^2^ = 0.036. Results of a TOST procedure with an epsilon of the standard deviation of the indoor condition were not significant (*mean difference* = 0.145; *epsilon* = 3.95; *confidence-interval*: −6.79 to 7.08; *df* = 7; *p* = 0.166).

For the positivity measure there was no significant main effect of stimulus, *F*_(1, 7)_ = 5.001, *p* = 0.060, η^2^ = 0.417. The main effect environment also was not significant, *F*_(1, 7)_ = 2.767, *p* = 0.140, η^2^ = 0.283. The interaction between stimulus and environment was significant, *F*_(1, 7)_ = 31.800, *p* = 0.001, η^2^ = 0.820. Amplitudes of the deviant trials were higher indoors (*M* = 9.54 μV, *SD* = 9.05 μV) than in the car (*M* = 5.18 μV, *SD* = 10.57 μV), while amplitudes in standard trials indoors (*M* = 0.02 μV, *SD* = 1.25 μV) were only slightly smaller than in the car (*M* = 0.92 μV, *SD* = 2.27 μV). Due to this significant interaction effect no TOST was performed.

Results from the t-test performed on mean peak latency differences of the indoor (*M* = 85 ms, *SD* = 46.3 ms) and the car condition (*M* = 101.5 ms, *SD* = 75.1 ms) were not significant (*p* = 0.569). The TOST procedure with an epsilon of the standard deviation of the indoor condition showed no significance for mean peak latency differences (*mean difference* = −16.5; *epsilon* = 46.3; *confidence-interval*: −68.8 to 35.8; *df* = 7; *p* = 0.158).

#### Induced alpha paradigm: frequency results

All individual correlation values for power spectral densities between conditions were higher than 0.79 on all five electrodes, with a mean correlation value of 0.97 (*SD* = 0.046). All *t*-tests of these correlations against zero were significant with *p*s < 0.0001. For the grand average, correlation values between indoor and car condition were both higher than 0.989, with a mean of 0.997 (*SD* = 0.004). *T*-tests against zero yielded significance (*p*s < 0.0001) for both conditions (engaged/relaxed).

The three-way repeated measures ANOVA with within-subject factors recording condition (*p* = 0.061), stimulus (*p* = 0.177), and electrode (*p* = 0.24) performed on mean alpha band powers was not significant on main or interaction effects, with non-significant interactions (all *p*s > 0.272). The TOST procedure with an epsilon of the standard deviation of the indoor condition assigned to mean alpha band powers showed significance on electrodes PO4 (*mean difference* = 0.049; *epsilon* = 0.129; *confidence-interval*: −0.031 to 0.128; *df* = 7; *p* = 0.049) and Oz (*mean difference* = 0.001; *epsilon* = 0.127; *confidence-interval*: −0.079 to 0.076; *df* = 7; *p* = 0.009). The TOST was not significant for electrodes PO3, POz, and Pz, all *ps* > 0.340.

**Figure 9:**
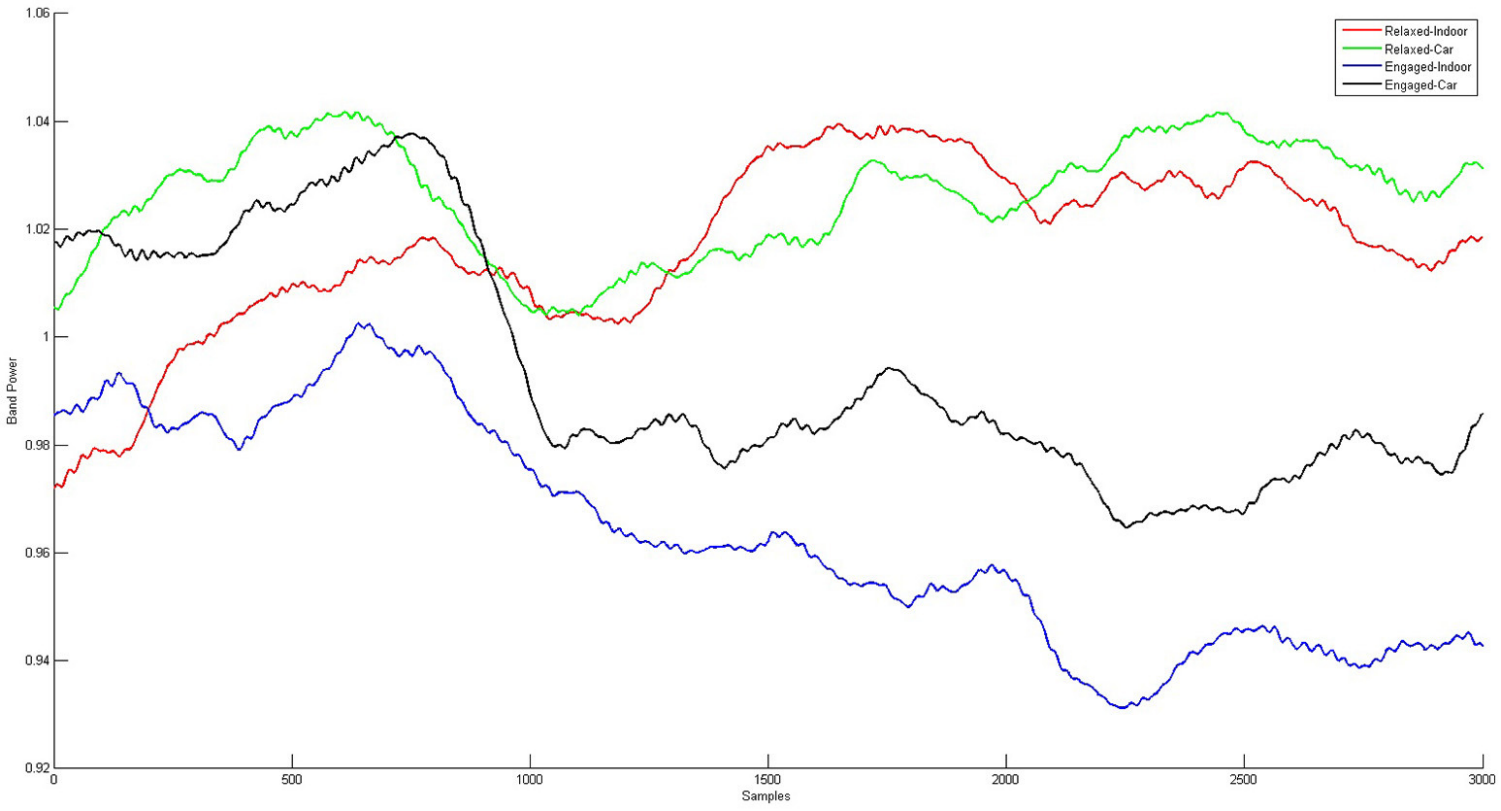
**Grand Averages of the alpha band time courses for relaxed and engaged conditions indoors and in the car**. For the red and the green curve, displaying the relaxed conditions, a similar pattern starting 1 s after onset of stimulus presentation is observed. Similarities over time are also apparent for the engaged conditions, represented in the black and blue curve. Clear co-variation of indoor and in car alpha time courses for both relaxed and engaged conditions is proven by high correlation between the signals.

Alpha band time course (see Figure 9) correlations between indoor and car condition yielded a mean correlation of *r* = 0.27 for the relaxed condition (Pz: *r* = 0.43, PO3: *r* = 0.26, PO4: *r* = 0.29, POz: *r* = 0.30, Oz: *r* = 0.09). Correlations in this condition were significant on all five electrodes for five participants (*p*s < 0.00001), on four electrodes for one participant (*p*s < 0.005), and for the other three participants on three electrodes (*p*s < 0.021). In the engaged condition the mean correlation of all participants was *r* = 0.23 (Pz: *r* = 0.34, PO3: *r* = 0.19, PO4: *r* = 0.18, POz: *r* = 0.31, Oz: *r* = 0.14). Tests yielded significance of correlations on all five channels for three participants (*p*s < 0.043). For three participants correlation was significant on four channels (*p*s < 0.00001) and for two participants on three electrodes (*p*s < 0.00001).

#### BCI results of both paradigms

A paired samples *t*-test indicated that the error rates for ERP classification in the indoor condition (*M* = 0.126, *SD* = 0.086) did not differ significantly from the error rates in the car condition (*M* = 0.145, *SD* = 0.116), *t*_(7)_ = −0.68149, *p* = 0.518. Furthermore, the TOST procedure with an epsilon of the standard deviation over participants in the indoor condition confirmed significant equivalence classification results in the two recording conditions (*mean difference* = 0.018; *epsilon* = 0.086; *confidence-interval*: −0.032 to 0.069; *df* = 7; *p* = 0.020).

A paired samples *t*-test indicated that the error rates of band power classification for the indoor condition was lower (*M* = 0.283, *SD* = 0.160), but did not differ significantly from the error rates in the car condition (*M* = 0.351, *SD* = 0.137), *t*_(7)_ = −1.608, *p* = 0.152. The TOST procedure with an epsilon of the standard deviation over the participants in the indoor condition confirmed significant equivalence for classification results in the two recording conditions (*mean difference* = 0.066; *epsilon* = 0.162; *confidence-interval*: −0.012 to 0.144; *df* = 7; *p* = 0.026).

### Block III: driving-related movements

**Figure 10:**
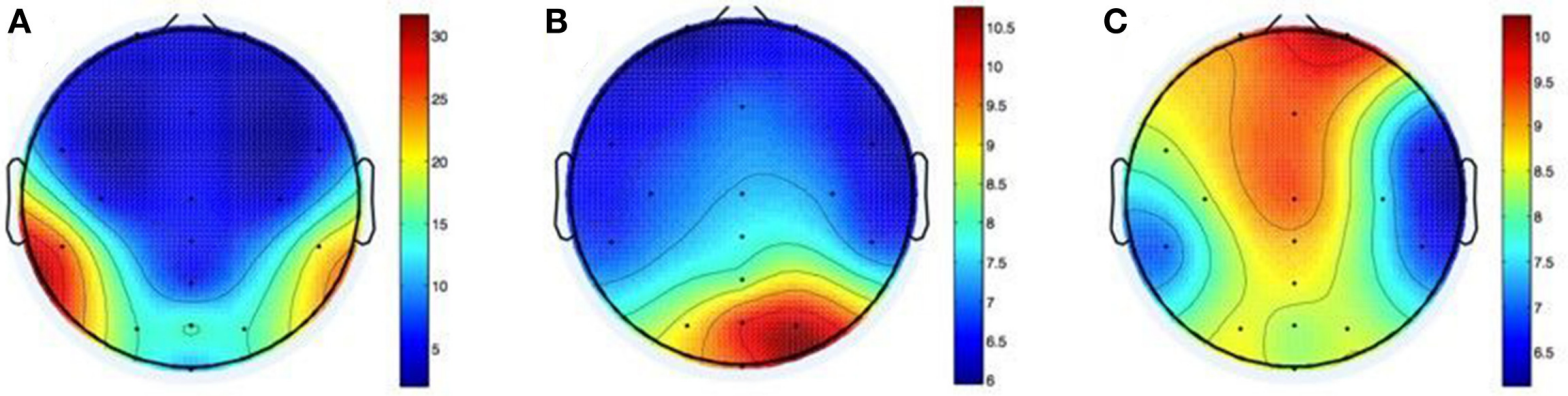
**Shifts in electrode positions after movements of the head (A)**, the arms **(B)**, and the whole body **(C)** in mm.

Figure 10 shows the shifts in electrode positions after each of the three groups of movements.

After head-related movements the difference between electrode positions (*M* = 9.6, *SD* = 9.1) differed significantly from zero, *t*_(9)_ = 3.3237, *p* = 0.009. The apparent lateralization of this effect (25.3 mm mean variation at CP5 vs. 19.6 mm at CP6) may be due to the direction of the shoulder check.

After performance of arm movements the mean difference between electrode positions (*M* = 7.6, *SD* = 4.8) differed significantly from zero, *t*_(9)_ = 5.0241, *p* = 0.001. Variations were located mainly to the right side of the head with a maximum of 10.5 mm mean variation at PO4. The cause for this may be the direction of the rotation and/or handedness of participants.

Mean electrode position differences after whole-body movements (*M* = 8.4, *SD* = 6.4) differed significantly from zero, *t*_(9)_ = 4.1691, *p* = 0.002. The greatest shift was on the forehead with 10.1 mm average variation on Fp2 and on the midline of the head (8.2 and 9.3 mm mean variation at POz and Fz). This could be caused by the cables, which were tied together, but interfered with the seatbelt nevertheless.

### Block VI: usability

The total SUS score of the system added up to 65. Following the official SUS score interpretation, this is slightly above the threshold for an acceptable system.

Due to minor delays during the experiments, the time points of the additional questionnaires varied slightly for each participant. On average, questions were answered after 60 (Block I), 122 (Block II), and 137.5 (Block III) min.

After the first 60 min, the system got a comfort rating of 7.5, which then decreased significantly over the next hour resulting in a rating of 3 after 122 min. In the following quarter of an hour needed for block III, the comfort rating stayed stable at 3. A Wilcoxon signed-rank test showed that there was a significant difference between the first time point of the rating after 60 min (*Mdn* = 7.5) and the second rating after 122 min (*Mdn* = 3), (*W* = 0, *Z* = −2.69, *p* = 0.008). No valid Wilcoxon signed-rank test could be performed to compare the second and third ratings, because the number of effective samples was less than 6 after subtraction of ratings equaled zero for six participants (*W* = 4, *Z* = −0.82, *p* = 0.625). Rating scores of the first and the third rating again showed significant differences, (*W* = 0, *Z* = −2.67, *p* = 0.008).

**Figure 11:**
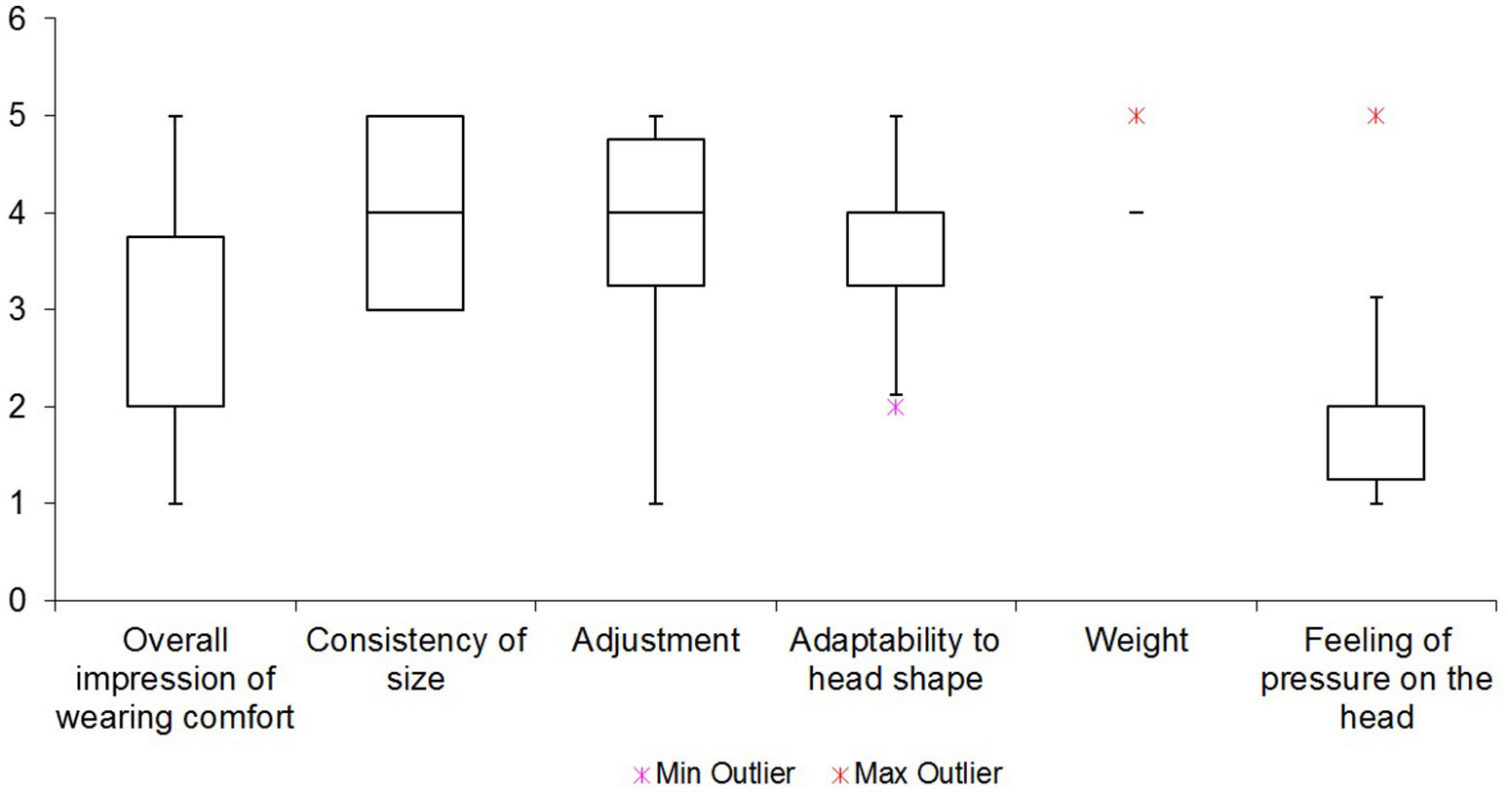
**Mean score of questions about wearing comfort**.

The six examined items of wearing comfort of the system are summarized in Figure 11. A feeling of pressure on the head was rated as the most irritating with a mean score of 2.2. The overall impression of wearing comfort got a mean score of 2.7, and was therefore also perceived as bad. The overall weight of the system on the head was on average rated as the most pleasant aspect of it with a score of 4.2.

Furthermore, the wearing comfort questionnaire yielded the following insights. Seven participants complained about dents and chafe marks on their heads, four about headaches, and one each about neck pains, nausea, and dizziness. Moreover, one participant had the subjective impression that the system had moved over the course of the experiments. None of the participants reported skin irritations due to wearing the cap.

## Discussion

### Block I: self-application

We found that the participants were equally fast as the experimenter in applying the cap, and equally capable in optimizing signal quality. We thus conclude that this type of dry electrode EEG system can indeed be used by individual end-users. We should note, however, that there was no objective measure of when the application was finished; it was based on individual judgements of the experimenter.

We did not investigate the personalization of the cap by adjusting the length of each electrode pin, because this task needs to be done only once. Therefore, we did not investigate how easy it is to personalize the cap while wearing it. Personalization did, however, take up quite some time. We assume that the QuickBit approach would benefit from improvement: Continuously adjustable bits would probably simplify personalization and optimize the result.

While it is not surprising that the signal quality was rated better with active display filters, we had assumed that the signal quality would be better after adjustments by an expert operator than compared to that adjusted by the participant. This, however, was not the case: Participants reached a similar, sometimes even better signal quality. We assume the reason for this to be that participants had a better feeling for how hard, and where exactly the electrodes pressed against their heads, allowing them to fit them even better to the scalp than the experimenter could without the risk of harming the participant.

For the electrode positions, some variation in the measurements must be taken into account. The used system has known variations in measured data points, and for some electrodes (primarily at the back of the head), the measuring stylus may have moved slightly due to head shifts that were sometimes necessary for the measurement. This problem was addressed mathematically, as described above. It was also not possible to point the stylus exactly at the electrode's point of contact with the skin, but only at the electrode's body. It remains unclear, whether or to what extent the differences in electrode positions we measured, imply that the points of contact changed as well.

### Block II: EEG recordings

For the oddball paradigm ERP analysis revealed highly similar morphology of ERPs elicited by deviant stimuli in both recording conditions. We found highly significant effects for the negative peak in the ERP condition. The deviant trials were significantly different from the standard trials in both the indoor and the car condition, showing no difference between conditions. This is not the case for the positivity. The main effect is not significant. It should be mentioned though that we have a clear tendency into the right direction with a p value slightly missing the threshold criteria of 5%. Peaks of the P300 are reduced in the car environment as a result of other signals interfering with the recorded signal in the car. No significant differences were found between peak latencies between indoor and car recordings. We conclude that the main information carried in the signal is comparable for indoor and in car recordings, but its signal strength is attenuated slightly in the car condition.

**Figure 12:**
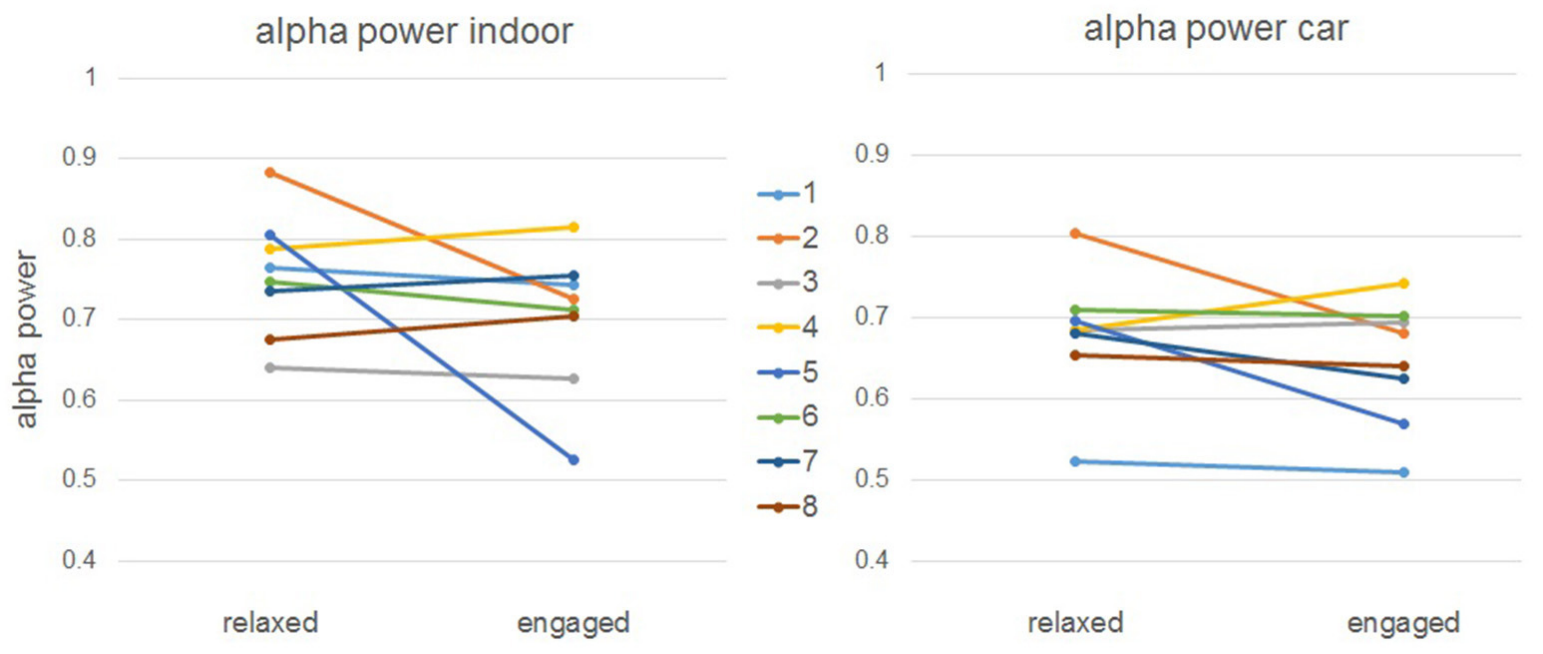
**Mean alpha power in relaxed and engaged trials for individual subjects**.

For the alpha recordings, we have a slightly more complex case. We clearly see a correlation between conditions—alpha values show a similar development over time outside of and in the car. However, there is no significant difference between relaxed and engaged trials on average over all participants, which was expected from the experimental design. When we take a closer look at the individual values (see Figure 12), we see that some participants managed to get relaxed in the corresponding task, while others did not. This explains why we do not get significant main effects—several participants were not able to relax in the appropriate condition. This effect can be seen consistently on both conditions, inside and outside the car. However, we do perhaps see a tendency on the main effect of condition that, even though it's not significant, indicates a small change in alpha power between recordings inside and outside of the car.

For all comparisons that showed no significant difference between conditions an equivalence test was performed. Features of the ERP were not equivalent between conditions while spectral features were equivalent on some of the tested electrodes.

These results show that even though we do not have significant differences, the recoded data cannot be taken equivalent. For strict neurophysiological measurements it hence might be worth a consideration whether the tested headset should be used or not.

For ERP and spectral data classifications were not significantly different, and were furthermore clearly equivalent. We, hence, assume that the evaluated system measured the differences in cognitive states, well, in both conditions. Despite small morphological and power differences, classification results were comparable in both domains. Therefore, a BCI can be applied with equal reliability to data from both conditions.

The results we found on the EEG components examined here are as expected from the literature and replicate results from a previous comparison study (Zander et al., 2011). Therefore, we conclude that the dry electrode system investigated here provides comparable data to a conventional gel-based system when used in an autonomous driving context.

It still remains unclear whether the results can be fully transferred to a real-world autonomous driving context where the car would most likely be moving. A driving car would bring additional factors like increased vibration from the engine, jerks due to uneven roads, or inertial effects induced by direction changes. Moreover, the driving task itself could lead to additional artifacts, such as stress related sweating on the scalp and the user scratching their own skin. Also head movements against the headrest might lead to changes of electrode positions in a way that was not examined here. Another factor would be the radio not being muted in a real-world-driving scenario: Environmental noises between 70 and 120 decibels have been found to increase the amplitude of measured P300 events (Nam et al., 2008). Drivers will also be moving e.g. their heads and hands, which they minimized during data recording. This study however presents a first step in investigating the applicability of dry systems in a car environment, revealing initial insights in a scenario with controlled artifact activity. These results can form the basis for future studies in active driving study scenarios, where that control is further relaxed.

### Block III: driving-related movements

The results showed that the electrodes shifted in position when executing different driving-related movements.

The most significant shifts occurred during movements involving the head directly, primarily at the rear left of the head. We assume this was due to the shoulder check, which required a sudden, fast turn of the whole head to the left and back. We can, however, not be sure as to whether the shoulder check or the look at the ceiling had more effect on the electrodes positions since they were measured together as one movement group. Either way, the resulting differences may well-influence the quality of the data recorded by the system.

The performed arm movements had less impact on the electrode positions, though the shifts were still significant.

The third group of movements resulted in the least position changes for all electrodes although the participants had to move their whole upper body—including the head. The most pronounced shifts were observed at the right frontal area. The instruction to touch the marker in the legroom of the passenger seat might offer an explanation for this, as the head had to be moved rather far to the right and down. Also in the area around the left ear increased shifts in position were observed. Most likely, this was a result of fastening and unfastening the seatbelt which may have induced some strain in that area, maybe by pulling on the cables.

Finally, since the movements were always performed in the same order (head, arm, and body), order effects cannot be excluded.

For future use, the cap could be applied e.g., only after the seat belt has been fastened, which often requires some effort. Since the cables may also have caused some of the position shifts, a wireless system is preferable.

### Block IV: usability

The *System Usability Scale* is a general questionnaire to evaluate the usability of technical systems, and is not specifically designed for BCI systems. As SUS provided significant insights in other BCI-related studies, we decided to use it here as well (Duvinage et al., 2012; Käthner et al., 2013). Some questions however, especially about the interaction with the system, did not fit the current purpose and even confused some of the participants. The resulting SUS score might therefore not be entirely accurate, but, we believe, still provides a good indication about the overall usability of the system in an autonomous driving context.

The evaluation of the wearing comfort was better tuned to the current context and raised no questions from participants. The results showed that the first hour of using the system did not bother the participants much, which qualifies it for short-term usage at least. After the second hour of using the system, however, the subjective comfort ratings dropped significantly and participants began to complain about dents, slight headaches, neck pain, even nausea and dizziness, which clearly shows that the EEG system with the current cap design is not suitable for long-term use. We did not investigate recovery time: How long a break is needed, before the cap can be comfortably worn again? This remains an open question.

The most annoying features of the system, according to the participants, were its rather tight fit onto the head resulting in the feeling of pressure. The overall weight of the system was, in contrast, rated to be quite pleasant which might be caused by the flexible, thin material of the cap. Also, participants rated the adaptability of the cap as quite high. The cap was rated as being fixated well, thanks to the chin belt and the holes for the ears providing a lot of stability–only one participant had the feeling the cap had moved at all.

## Conclusion

Concluding in brief, the EEG system allowed for technically sound recordings, even with car-induced interferences present. It thus appears to be suitable for passive BCIs in autonomous driving scenarios, allowing mental states to be detected in real time.

In only a few minutes, individuals were able to apply and adjust a pre-customized cap, with the help of a little mirror, like the rear view mirror of a car. A system to better support the evaluation of signal quality would be beneficial, however.

According to the system usability scale, the system is at the edge of acceptability in terms of usability. This may suffice for professional drivers, who likely stand to gain the most from autonomous driving and supportive systems, but room for improvement remains. In particular the reported discomfort after longer use is unacceptable. Here, major improvement is necessary to decrease pressure on the scalp so the system is no longer obstructive and uncomfortable, hindering the users from focusing on themselves and their tasks.

Seeing now that EEG technology has made clear progress toward ease of use and mobile scenarios, we can envision the application of passive BCIs in the context of autonomous driving. Passive BCIs can provide essential information about the driver's cognitive or affective state, which can be combined with other sensor data of the car. In that way, the car can adapt to, and make decisions informed by, individual aspects of the driver. As passive BCIs do not rely on directed or even conscious actions of the driver (Zander and Kothe, 2011), the car will still drive autonomously but gains an additional stream of information, pertaining to the subjective situational interpretation of the driver.

For example, we can clearly imagine applications improving safety and comfort. In cases where the driver is required to take over control, the communication of this requirement can be adapted to the current, actual state of the driver. Another scenario would be the detection of whether or not communicated alarm signals were perceived and processed by the driver. These are only a few, simple examples of a broad range of applications to be thought of.

Moreover the investigated system could be used in a broader field of scenarios and might be of special interest for the field of Mobile brain/body imaging (MoBI). The field's objective is to acquire neurophysiological recordings of human cognition in real world environments where subjects perform real-world tasks. A portable, wireless, high-quality data recording and fast to prepare dry contact system would prove useful for brain activity recordings on actively behaving participants (Gramann et al., 2011, 2014; De Sanctis et al., 2012).

The application of passive BCI during autonomous driving however provides an exemplary use case for technology that adapts to the (neuronal) state of its operator during automation in general. Such Neuroadaptive Technology is a clear additional step toward closing the cybernetic loop (Pope et al., 1995).

## Ethics statement

The study involved standard EEG procedures covered in an ethic statement approved by the ethics committee of the Institute of Psychology and Ergonomics of the Berlin Institute of Technology. All participants gave written consent to their participation in the conducted study. They were provided with information on the purpose of the study, given the opportunity to ask questions and were informed that their participation was voluntary and they could end the experiment whenever they liked without a need to provide reasons. Participants also gave their consent for data recording, anonymous storage of that data, as well as its usage for publication.

## Author contributions

All authors contributed substentially to the work presented here. Everybody was contributing to the drafting and revising of the documents and approved the final version. Everybody agreed to be accountable for the integrity and accuracy of the work. Specifically: TZ designed and supervised the experimental procedures, conducted and supervised the analyzes, interpreted the results for the context of autonomous driving. LK and KG were responsible for quality of writing and validation of results. Everybody below was involved in conducting the experiments and ensured data quality. LA was responsible for the statistical analyzes and integrity of the manuscript. JP and MB were responsible for the electrode localization and the related mathematical procedures. LZ: Was responsible for the programming and EEG and BCI analyzes. AB: Was responsible for evaluation of the questionnaires.

### Conflict of interest statement

The authors declare that the research was conducted in the absence of any commercial or financial relationships that could be construed as a potential conflict of interest.

## Footnotes

^1^Simulation and Neuroscience Application Platform (SNAP). Available: https://github.com/sccn/SNAP.
